# Data set on the synthesis and properties of 2′,3′-dideoxyuridine triphosphate conjugated to SiO_2_ nanoparticles

**DOI:** 10.1016/j.dib.2018.09.127

**Published:** 2018-10-04

**Authors:** Svetlana V. Vasilyeva, Inga R. Grin, Boris P. Chelobanov, Dmitry A. Stetsenko

**Affiliations:** Institute of Chemical Biology and Fundamental Medicine, SB RAS, 8 Lavrentiev Avenue, Novosibirsk 630090, Russia

**Keywords:** Cellular delivery, Click-chemistry, Phosphorylated nucleosides, MCF7 cells, Cytotoxicity

## Abstract

SiO_2_ nanoparticles were used as a transport system for cellular delivery of phosphorylated 2′,3′-dideoxyuridine to increase its anticancer potency. This data set is related to the research article entitled “2′,3′-Dideoxyuridine triphosphate conjugated to SiO_2_ nanoparticles: synthesis and evaluation of antiproliferative activity” (Vasilyeva et al., 2018) [1]. It includes a protocol for the synthesis of 2′,3′-dideoxyuridine-5′-{N-[4-(prop-2-yn-1-yloxy)butyl]-γ-amino}-triphosphate, its identification by NMR, IR and ESI-MS, experimental procedure of covalent attachment to SiO_2_ nanoparticles with *via* Cu-catalyzed click-chemistry, experimental data on chemical stability of the conjugate at different pH values and cytotoxicity assessment of antiproliferative effect of the conjugate.

## Specifications table

**Table:** 

Subject area	*Bioсhemistry, Chemistry, Nanobiotechnology*
More specific subject area	*Synthesis of phosphorylated nucleosides and their conjugation to SiO*_*2*_*nanoparticles*
Type of data	*Experimental synthesis protocols, Tables, Figures, Text file*
How data was acquired	*NMR, IR, mass spectrometry, analytical HPLC, MTT assay*
Data format	*Raw, Analyzed*
Experimental factors	*γ-Alkynyl 2*′*,3*′*-dideoxyuridine triphosphate was synthesized and characterized. A conjugate of the triphosphate with SiO*_*2*_*nanoparticle was then obtained by click-chemistry. Chemical stability of the conjugate at different pH values was studied and its cytotoxicity for MCF7 cells analyzed.*
Experimental features	*Experimental details of the synthesis of 2*′*,3*′*-dideoxyuridine triphosphate conjugated to SiO*_*2*_*nanoparticles; data on chemical stability and antiproliferative activity of the conjugate.*
Data source location	*Novosibirsk, Russia*
Data accessibility	*The data are available with this article*
Related research article	*This data set is submitted as a companion paper to the research article:* Svetlana V. Vasilyeva, Inga R. Grin, Boris P. Chelobanov, Dmitry A. Stetsenko. 2′,3′-Dideoxyuridine triphosphate conjugated to SiO_2_ nanoparticles: synthesis and evaluation of antiproliferative activity. *Bioorg Med Chem Lett.* 2018; 28:1248, DOI: 10.1016/j.bmcl.2018.02.007.

## Value of the data

• The paper relates to the research area of nanoparticle-based biomaterials for biomedical and bioengineering applications.
• This data set presents a protocol for synthesis and characterization of a new bionanoconjugate, which could be used by researchers in the same area.
• The data include a preliminary assessment of antiproliferative properties of a novel conjugate of 2′,3′-dideoxyuridine triphosphate with SiO_2_ nanoparticles.

## 1 Data

**Fig. 1:**
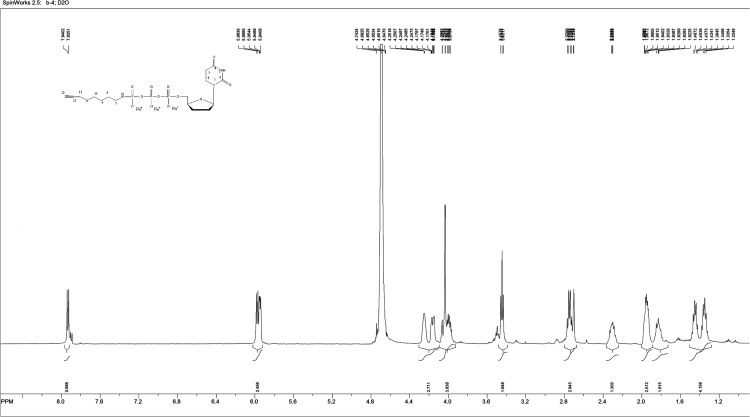
^1^H NMR spectrum of L~pppddU.

**Fig. 2:**
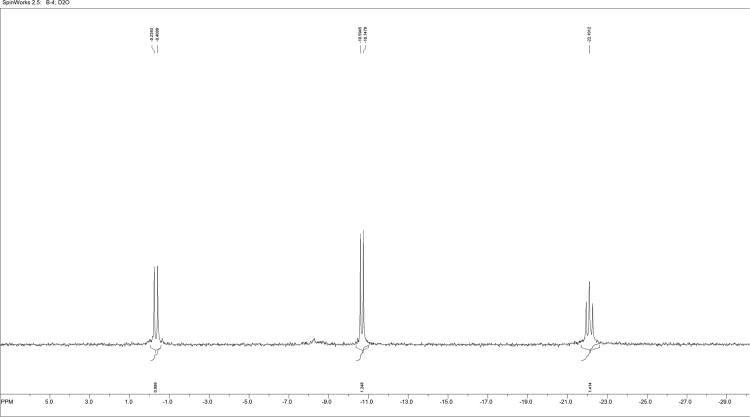
^31^P NMR spectrum of L~pppddU.

**Fig. 3:**
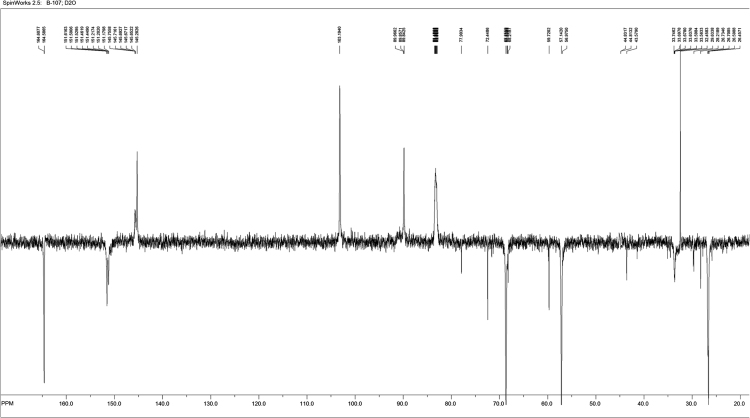
^13^C NMR spectrum of L~pppddU.

**Fig. 4:**
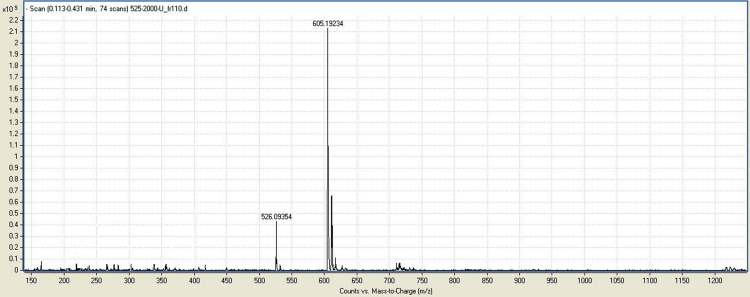
ESI-MS of L~pppddU. Calc. for [M+H-Na]^+^ 605.29 *m*/*z*, found 605.19.

**Fig. 5:**
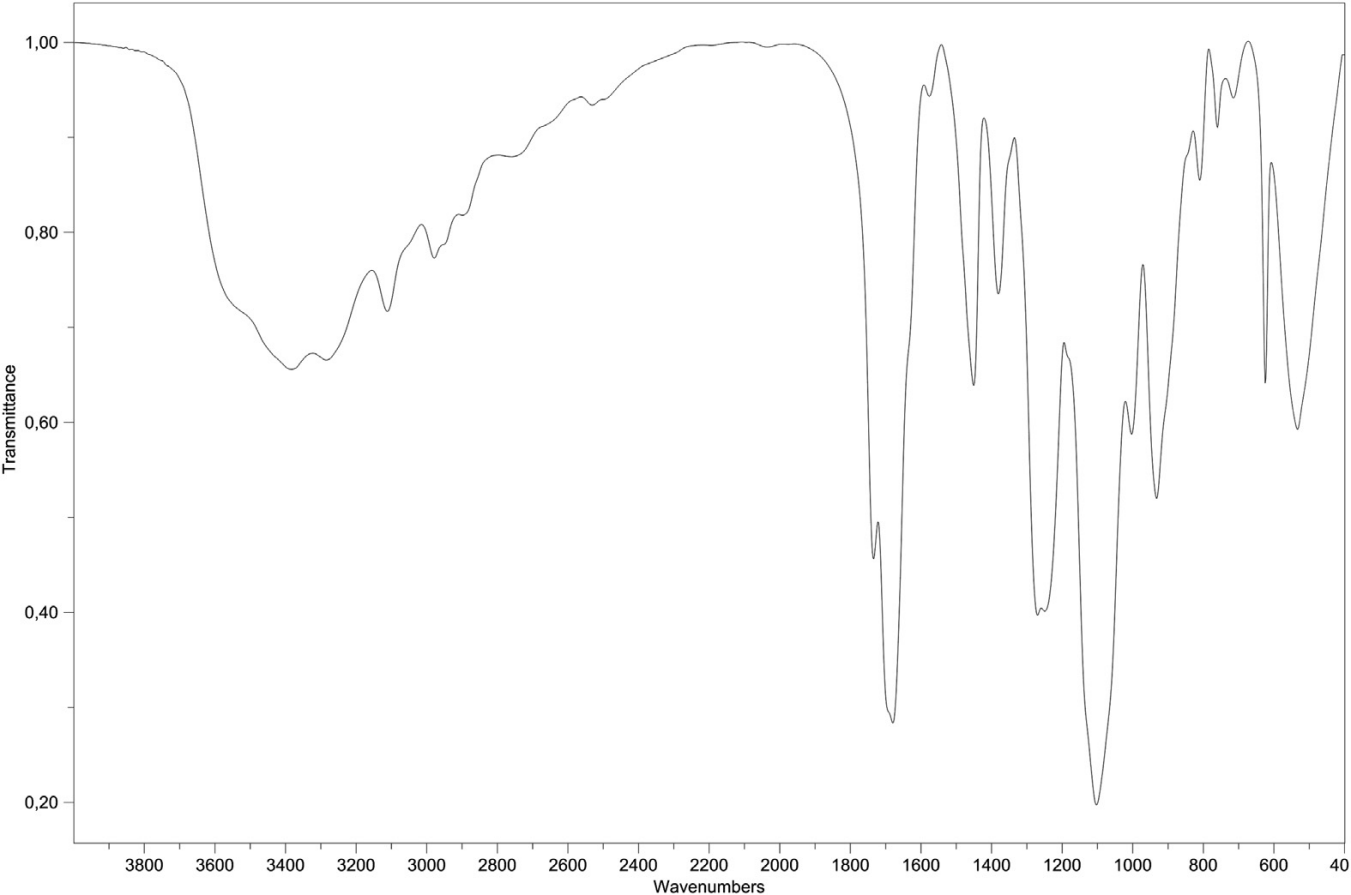
IR spectrum of L~pppddU.

Data set includes experimental conditions for the synthesis of 2′,3′-dideoxyuridine-5′-{*N*-[4-(prop-2-yn-1-yloxy)butyl]-γ-amino}-triphosphate and its characterization by ^1^H, ^13^C, and ^31^P NMR, IR and ESI-MS (Fig. 1, Fig. 2, Fig. 3, Fig. 4, Fig. 5), and protocol for its click-chemistry conjugation to azido-modified SiO_2_ nanoparticles [1], [2]. Experimental data on chemical stability of the conjugate at different pH values are presented (Table 1, Table 2). Cytotoxicity of the conjugate for MCF7 cell line has been assessed (Table 3).

**Table 1:** Preparation of sample and control solutions.

No. sample	Compound	Concentration, mM			Nucleotide content *ν*, µM/mg
		pH 7.3 (PBS)	pH 6.5 (P)	pH 1.5–2 (HCl)	
1	ddU	37.7	28.2	61.2	MW 212.21
2	pppddU	28.8	15.4	34.6	MW 520.27
3	L~pppddU	31.1	44.9	20.0	MW 579.09
5	SiO_2_~L*~pppddU	8	8,8	8	0.400

**Table 2:** Stability of SiO_2_~L*~pppddU conjugate and controls at different pH values.

*N* sample	Sample	Loss of nucleotide content in the sample^a^ after 48 h, %			Nucleotide content, *ν* µM/mg
		pH 7.3 (PBS)	pH 6.5 (P)	pH 1.5–2 (HCl)	
1	ddU	0	0	0	–
2	pppddU	2	0	7	–
3	L~pppddU	9	14	51	–
5	SiO_2_~L*~pppddU	36	40	6	0.400

a Loss of nucleotide content in the samples (1–3) due to decomposition of the controls.

**Table 3:** IC_50_ values for MCF7 human breast adenocarcinoma cell line (means ± standard deviations).

Compounds	IC_50_, (MTT),
SiO_2_~L*~pppddU	183 ± 57 μg/mL or 22 ± 0.7 μM
SiO_2_~L*	no inhibition
ddU	no inhibition
pppddU	no inhibition
L~pppddU	no inhibition

## 2 Experimental design, materials, and methods

### 2.1 Materials and methods

Reagents and solvents were purchased from Sigma-Aldrich or Acros Organics, and used without purification. Aminoalkylated SiO_2_ nanoparticles were from SkySpring Nanomaterials (USA). Mass spectra were obtained on an Agilent 6410 Triple Quadrupole LC-MS device (USA).^1^H, ^13^C and ^31^P NMR spectra were recorded using Bruker AV-400, AV-300 or DRX-500 NMR spectrometers. UV absorption spectra were obtained on a Shimadzu UV-1800 spectrophotometer (Japan). Analytical HPLC was carried out on an Agilent 1200 Series LC system, using Zorbax^®^ 5 µm Eclipse-XDB-C18 80 Å column 150 × 4.6 mm by Agilent (USA).

### 2.2 Synthesis of 2′,3′-dideoxyuridine-5′-{*N*-[4-(prop-2-yn-1-yloxy)butyl]-γ-amino}-triphosphate (L~pppddU)

2′,3′-Dideoxyuridine-5-triphosphate ammonium salt (pppddU) was synthesized according to [3]. NMR data were in good agreement with the literature [4]. 1-Ethyl-3-(3-dimethylaminopropyl)carbodiimide hydrochloride (EDC·HCl) (368 mg, 1.92 mmol, 5 equiv.) was dissolved in deionized water (4 mL), pppddU (200 mg, 0.384 mmol, 1.0 eq) was added and the pH was adjusted to 7.5 with aq NaOH (0.1 M, 0.6 mL). After stirring at ambient temperature for 7 min, 3-aminobutyl propargyl ether (220 µg, 0.844 mmol, 2.2 eq) was added. After complete consumption of the starting material according to RP-HPLC, the mixture was precipitated with 6% LiClO_4_ in acetone. Crude product was purified by anion exchange chromatography on DEAE Sephadex A-25 column eluted with a linear gradient from 20% EtOH to 1 M NH_4_HCO_3_ in 20% EtOH. Fractions were collected and evaporated to dryness, the residue was co-evaporated several times with 96% EtOH and the product was precipitated with 6% NaClO_4_ in acetone to yield 100 mg (45%) of the γ-alkynyl triphosphate, L~pppddU. ^1^H NMR (500 MHz, D_2_O): *δ* (ppm) 7.9 (*d*, 1H, H(6)-(U), *J* = 7.7 Hz); 5.98–5.94 (*m*, 2H, H(5)-(U), H1′); 4.25–4.17 (*m*, 2H, H4′, H5′); 4.06–3.96 (*m*, 3H, H5′, OC*H*_2_C≡CH); 3.44 (*t*, 2H, OC*H*_2_(CH_2_)_3_NH, *J* = 6.5 Hz); 2.77–2.70 (*m*, 3H, O(CH_2_)_3_C*H*_2_NH, C*H*≡C); 2.34–2.27 (*m*, 1H, H2′); 1.97–1.93 (*m*, 3H, H3′, 2′); 1.84–1.83 (*m*, 1H, H3′); 1.49–1.32 (*m*, 4H, OCH_2_(C*H*_2_)_2_CH_2_NH). ^13^C NMR (75 MHz, D_2_O): *δ* (ppm) = 164.6 (4-C), 151.6 (2-C), 145.7 (6-C), 103.2 (5-C), 89.8.19 (1′-C), 82.9 (4′-C), 77.9 (12-C), 72.4 (10-C), 68.7 (11-C), 59.7 (5′-C), 57.1 (7-C), 43.6 (2′-C), 33.6 (3’-C), 29.6 (13-C), 26.7 (8-C), 26.6 (9-C). ^31^P NMR (121 MHz, D_2_O): *δ* (ppm) = − 0.3 (*d*, *J* = 20.69 Hz, *γ*-P), −10.74 (*d*, *J* = 18.69 Hz, *α*-P), − 22.10 (*t*, *J* = 20.03 Hz, *β*-P). UV spectrum (0.2 M NaOH): λ_max_ 260 nm, ε 1883. IR (cm^−1^): 3360, 3276, 3100, 2973, 2877, 2493, 1660, 1524, 1430, 1385, 1223, 1100, 995, 906, 810, 767. ESI-MS: [M+H-Na]^+^calcd for C16H24N3O13P3Na2=605.29, found 605.19.

### 2.3 Synthesis of SiO_2_~L*~pppddU and control SiO_2_~L^*^conjugates by CuAAC reaction (Fig. 6)

**Fig. 6:**
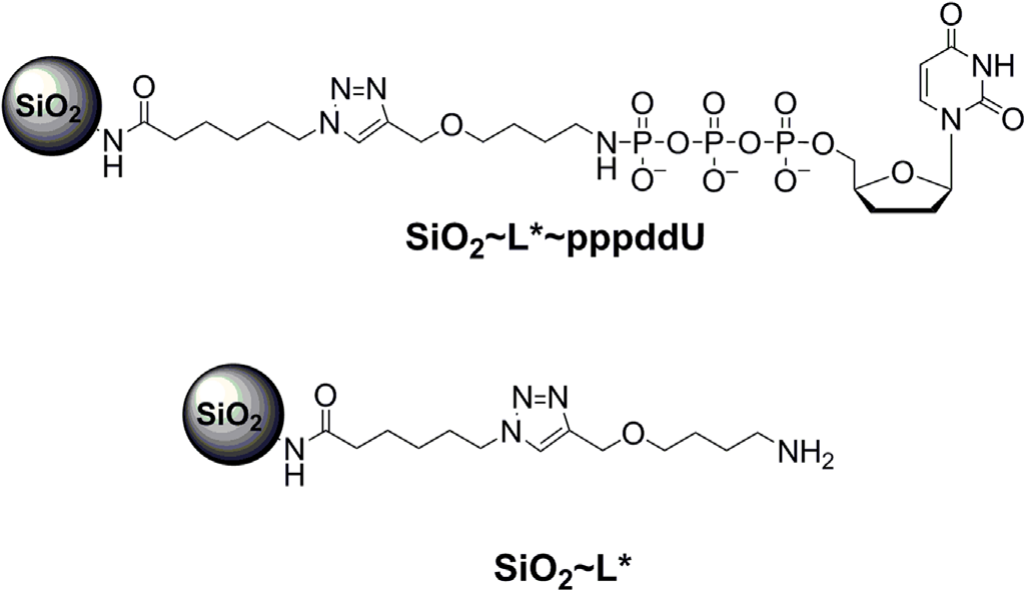
Structure of the conjugate of 2′,3′-dideoxyuridine triphosphate with SiO_2_ nanoparticles (SiO_2_~L*~pppddU) and control unloaded conjugate (SiO_2_~L*).

Azido-modified SiO_2_ nanoparticles [2] (40 mg, 17.2 µmol) were sonicated in 200 µL of H_2_O. A solution of L~pppddU (100 mM in H_2_O, 1.386 mL, 139 µmoL, 8 equiv.) was added to the suspension followed by copper(II) sulfate (172 µL of 0.5 M solution in water), 1 M trietylammonium acetate (TEAAc) buffer, pH 7 (172 µL) and sodium ascorbate (86 µL of freshly prepared 1 M solution in water). The reaction mixture was purged with argon for 2 min and stirred at ambient temperature for 12 h. Conjugate was separated by centrifugation, supernatant was discarded. Precipitate was washed successively with 0.1 M NaCl, 10% Na_2_EDTA, water, diethyl ether followed by air drying. Yield: 38.5 mg. The control unloaded SiO2~L*~conjugate was obtained as described above, but without addition of triphosphate. Yield: 9 mg. IR: N_3_ group band was absent. The nucleotide content of the conjugate was measured by UV absorbance after dissolving in 0.2 M NaOH. UV spectrum of L~pppddU (0.2 M NaOH): λ_max_ 260 nm, ε 1883. Nucleotide content was calculated as UV absorbance of SiO_2_~L*~pppddU conjugate minus UV absorbance of SiO_2_~L* conjugate: 0.4 µmol/mg (Fig. 6).

### 2.4 Hydrolysis of SiO_2_~L*~pppddU conjugate at different pH

The conjugate (0.400 µM/mg nucleotide) was dispersed in three buffers with pH values corresponding to the pH in the blood (pH 7.3), the mouth (pH 6.5) and the stomach (pH 1.5–2.0) (2 mg in 100 µL of each buffer). Parent nucleoside (ddU), its 5’-triphosphate (pppddU) and alkynyl triphosphate (L~pppddU) solutions in the same buffers were prepared (Table 1). The solutions were incubated at 37 °C in a thermomixer. Initial aliquot (15 μL) was taken and analyzed by analytical HPLC at the wavelength 260 nm. Appropriate aliquots of buffers (15 μL) after centrifugation were analysed after 1, 4, 24 and 48 h. Percentage loss of the nucleotide content in the sample of SiO_2~_L*~pppddU was evaluated at the end of the experiment. The remaining buffers were discarded after centrifugation; conjugate was washed successively with water followed by dissolution in 0.2 M NaOH and UV measurement of the remaining nucleotide content.

### 2.5 Cytotoxicity assay for human breast adenocarcinoma MCF7 cells

Cells were grown as adherent monolayer cultures in T25 cm^2^ culture flasks in complete Dulbecco׳s Modified Eagle׳s Medium (DMEM) supplemented with 10% fetal bovine serum, 1 mM sodium pyruvate, 2 mM L-glutamine and 1% penicillin-streptomycin-amphotericin B. Cultures were grown at 37 °C under a humidified atmosphere of 5% CO_2_ at 37 °C. Cytotoxicity was determined by the colorimetric MTT assay [5]. Cells were harvested from culture flasks by trypsin treatment and seeded in complete medium (100 μL/well) into 96-well plates at 1.5 × 104 cells per well. The cells were allowed to settle and resume proliferation for 24 h. Stock solutions of test compounds were prepared in DMEM, diluted by complete medium and immediately added to the plates (100 μL/well). Control groups with untreated cells were grown under the same condition in complete medium. After 72 h the medium was removed and replaced with 100 μL/well of 10% MTT solution in complete medium, and the cells were incubated for 3 h at 37 °C. The supernatant was removed, 100 μL of isopropanol was added, and the plates were gently shaken to solubilize the formazan crystals formed. Absorption was measured with a microplate reader (Multiscan EX, Thermo Fisher Scientific, Waltham, MA) at 570 nm and a reference wavelength of 620 nm. The cytotoxicity was expressed as cell growth inhibition (GI) at each group of the cells [6], [7]. All samples were analyzed using at least three independent experiments with triplicates for each concentration. The results are shown in Table 3.
